# Single amino acid bionanozyme for environmental remediation

**DOI:** 10.1038/s41467-022-28942-0

**Published:** 2022-03-21

**Authors:** Pandeeswar Makam, Sharma S. R. K. C. Yamijala, Venkata S. Bhadram, Linda J. W. Shimon, Bryan M. Wong, Ehud Gazit

**Affiliations:** 1 Department of Chemistry, Indian Institute of Technology (BHU), Varanasi, UP 221005 India; 2 Department of Chemical & Environmental Engineering and Materials Science & Engineering Program, University of California-Riverside, Riverside, CA 92521 USA; 3 Department of Chemistry, Indian Institute of Technology Madras, Chennai, 600036 India; 4 Center for Atomistic Modelling and Materials Design, IIT Madras, Chennai, 600036 India; 5 IMPMC, Sorbonne Université, CNRS, MNHN, 4, place Jussieu, 75005 Paris, France; 6 Chemical Research Support, The Weizmann Institute of Science, 7610001 Rehovot, Israel; 7 The Shmunis School of Biomedicine and Cancer Research, Tel Aviv University, Tel Aviv, 6997801 Israel; 8 Department of Materials Science and Engineering, Iby and Aladar Fleischman Faculty of Engineering, Tel Aviv University, Tel Aviv, 6997801 Israel; 9 Present Address: Division of Sciences, Krea University, Sri City, 517646 India

**Keywords:** Biocatalysis, Environmental monitoring, Biocatalysis, Two-dimensional materials, Biosensors

## Abstract

Enzymes are extremely complex catalytic structures with immense biological and technological importance. Nevertheless, their widespread environmental implementation faces several challenges, including high production costs, low operational stability, and intricate recovery and reusability. Therefore, the de novo design of minimalistic biomolecular nanomaterials that can efficiently mimic the biocatalytic function (bionanozymes) and overcome the limitations of natural enzymes is a critical goal in biomolecular engineering. Here, we report an exceptionally simple yet highly active and robust single amino acid bionanozyme that can catalyze the rapid oxidation of environmentally toxic phenolic contaminates and serves as an ultrasensitive tool to detect biologically important neurotransmitters similar to the laccase enzyme. While inspired by the laccase catalytic site, the substantially simpler copper-coordinated bionanozyme is ∼5400 times more cost-effective, four orders more efficient, and 36 times more sensitive compared to the natural protein. Furthermore, the designed mimic is stable under extreme conditions (pH, ionic strength, temperature, storage time), markedly reusable for several cycles, and displays broad substrate specificity. These findings hold great promise in developing efficient bionanozymes for analytical chemistry, environmental protection, and biotechnology.

## Abstract (web summary)

In the context of enviromental applications, refining enzymes into more minimalist structures could ease production costs, improve stability, and improve reusability. Here, the authors report a single amino acid bionanozyme that can catalyze the rapid oxidation of environmentally toxic phenolic contaminates and serves as a tool to detect biologically important neurotransmitters similar to the laccase enzyme.

## Introduction

**Fig. 1:**
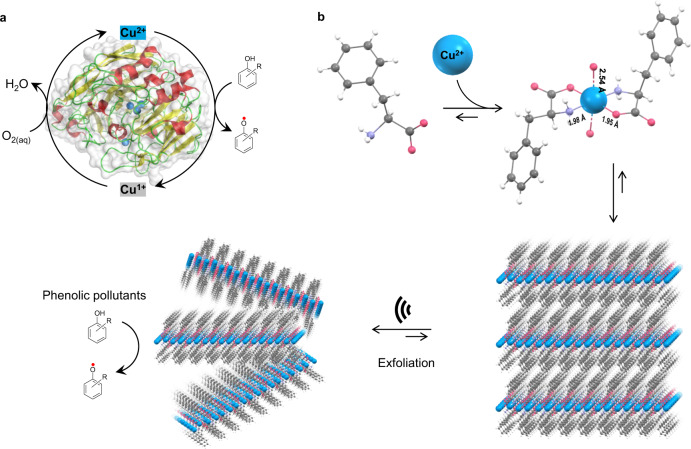
Bioinspired design of a laccase-mimicking F-Cu 2D layered bionanozyme. **a** Structure of natural Laccase (PDB entry 1V10), comprised of a redox (Cu^2+^/Cu^1+^) multicopper-coordinated catalytic site involved in the catalytic oxidation of toxic phenolic compounds into phenoxy radicals via reduction of oxygen to water. **b** Chemical structure and anticipated schematic illustration of F-Cu nanozyme formation. F spontaneously coordinates with Cu^2+^ via carboxylate amine chelating modes into a hierarchical 2D van der Waals layered F-Cu structure. Further mechanical (ultrasonication) exfoliation to F-Cu monolayers facilitates laccase-mimicking oxidation of environmentally toxic phenol.

Over billions of years, enzymes naturally evolved to efficiently catalyze various thermodynamically challenging biochemical reactions necessary to sustain life under physiological conditions^1^. Nonetheless, the exact evolutionary pathway from the simple amino acid building blocks to complex enzyme structures is still enigmatic^2–4^. Enzymes also serve as very useful biocatalysts in several industrial processes due to their numerous advantages over conventional organic or inorganic catalysts, including milder reaction conditions, exceptional selectivity, and lower environmental and physiological toxicity^5–8^. Many oxidative enzymes have been studied that could potentially transform many environmentally toxic compounds into less harmful products^9,10^. Among these, laccases are multicopper-containing oxidases commonly found in bacteria, fungi, plants, and insects that have raised significant interest in both fundamental and applied research^11^. These enzymes catalyze the oxidation of a wide array of environmentally toxic phenolic contaminants into unstable phenoxy radical intermediates that further self-couple to form benign polymeric substances, highlighting their use in biotechnology and environmental remediation (Fig. 1a)^12^. Unlike other oxidase enzymes that require or produce hydrogen peroxide, laccase utilizes only molecular oxygen as the final electron acceptor and releases water as a by-product, with the copper redox (Cu^2+^/Cu^1+^) reactivity playing a critical role in shuttling the electrons from substrates to oxygen. Hence, laccases are considered eco-friendly and versatile biocatalysts with enormous potential for industrial wastewater treatment. Although laccases are very promising, several intrinsic drawbacks, including high costs of preparation and purification, low operational stability, sensitivity of catalytic activity to environmental conditions, and inefficient recycling and reuse, severely hamper their widespread use for in vitro applications.

One promising approach to overcome the limitations of applying natural enzymes in industrial settings is to utilize enzyme-mimicking nanomaterials, termed nanozymes^13,14^. The concept of nanozymes has emerged as the next generation of enzymes since it links nanomaterials to biological systems and their functions^15^. Compared to natural enzymes, nanozymes are highly cost-effective for production and quite robust for diverse applications. Consequently, numerous classes of nanomaterials, including metal oxide nanoparticles, metal nanoparticles, transition metal chalcogenides, alloy nanomaterials, carbon-based nanomaterials, graphene quantum dots, carbon nitride nanomaterials, and metal-organic frameworks have been discovered to possess unique enzyme-mimetic catalytic activities^14,16–18^. However, it should be noted that most of these materials do not share any common properties or structures of the native enzymes and suffer from harsh synthesis conditions, complicated fabrication steps, sophisticated equipment, and time-consuming and hard-templating methods, thus greatly impeding their use in industrial-scale applications. In this regard, peptide-based nanomaterials have attracted growing attention due to their protein-derived nature, ease of large-scale synthesis, and good biodegradability^19–21^. In the past few years, the minimalist approach originally described by DeGrado as “sequences that are simpler than their natural counterparts but, nevertheless, retain sufficient complexity for folding and function” has materialized in the construction of simple biomolecular nanozymes (bionanozymes)^2,22–34^. Despite many efforts, the de novo design and development of laccase mimicking bionanozymes is still in its infancy owing to the complex structure of the laccase active site and the complicated catalytic reaction mechanism.

Moving even more minimalistic, in this work, we explored whether the self-assembly of a single amino acid can produce a laccase-mimicking bionanozyme for environmental remediation. Inspired by the laccase catalytic active site, we explore the supramolecular assembly of the single amino acid phenylalanine (F) in combination with redox-active divalent copper ions (Cu^2+^). Among the pool of amino acids, F is an excellent self-assembling structural unit containing metal-chelating amino and carboxylate functionalities; therefore, it acts as a multidentate ligand to coordinate with the divalent Cu^2+^ ^35–37^^,^. The envisioned supramolecular organization of F in the presence of Cu^2+^ is shown in Fig. 1b, where F coordinates with Cu^2+^ to form a two-dimensional (2D) layered structure that is stacked together through aromatic interactions (F-Cu). The relatively weak van der Waals interlayer interactions facilitate mechanical exfoliation into either monolayers or several-layer-thick F-Cu nanosheets. The ultrathin 2D nanosheets coupled with highly accessible crystalline 2D arrays of redox Cu^2+^/Cu^1+^ ions are anticipated to exhibit an enhanced laccase mimicking catalytic oxidation of toxic phenolic compounds with exceptional stability. Therefore, this single-amino acid bionanozyme approach represents a potential alternative to both protein engineering and other conventional nanozyme approaches.

## Results

### Synthesis and structural analysis of the F-Cu bionanozyme

**Fig. 2:**
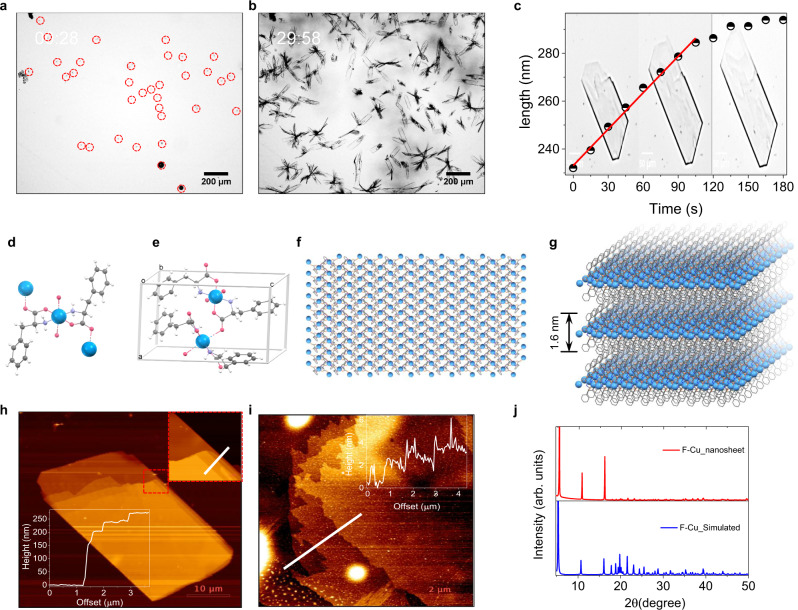
Structural characterization of F-Cu. **a**, **b** Optical microscopy snapshots taken from the in situ real-time monitoring of the F-Cu crystallization process (Supplementary Movie 1), showing the formation of **a** small nucleation seeds (red dotted circles) at 28 s and **b** grown micrometer-long 2D crystals at ~30 min. Each experiment was repeated three times independently with similar results. **c** Optical microscopy snapshots are taken from Supplementary Movie 2, which monitors the in situ crystallization kinetics of the F-Cu 2D layered crystals and corresponding graphical representations of the elongation rate. The calculated average measured elongation rate is 7.89 nm s^−1^. **d**–**g** Single-crystal X-ray structure of F-Cu (**d**), F-Cu assembly exhibits an octahedral primary coordination complex structure (**e**). The unit cell is comprised of four F molecules and two Cu^2+^ ions (**f**), 2D infinite layered F-Cu coordination complex structure shown along the crystallographic plane. **g** Hierarchical 2D van der Waals layered stacked structure shown along the crystallographic plane. **h**, **i** AFM images and height profiles of F-Cu 2D layered crystals **h** before and **i** after ultrasonication treatment, displaying the mechanical exfoliation of pristine 2D hierarchical layered F-Cu crystals into single or several-layered nanosheets. The AFM height profiles of nanosheets (~1.8 nm thick) are in good agreement with the single atomic layer thickness in the crystal structure (~1.6 nm thick, **g**). **j** PXRD patterns simulated from F-Cu crystal structure and experimental 2D nanosheets.

Mixing of Cu^2+^ with an F alkaline solution under mild heating conditions resulted in the formation of blue-colored F-Cu 2D plate-like crystals, which were analyzed using single-crystal X-ray crystallography (Fig. 2). The coordination of Cu^2+^ with F, shown in Fig. 2d, was octahedral with tetragonal distortion. Two F molecules complexed with one Cu^2+^ through O (carboxylate) with N (amine)-chelating modes, forming two five-membered phenylalanate-Cu^2+^ chelate rings. The two aromatic phenyl rings on either side of the chelating rings were in a *trans* conformation with respect to each other. These primary coordination spheres were further linked together by the second oxygen atom of the carboxylate groups of both F molecules and thus formed an infinite in-plane, strongly-coordinated, covalent 2D sheet along the crystallographic *a* and *b*-axis (Fig. 2e, f). Further, these atom-thin 2D sheets are stacked into a layer-by-layer assembly held together through relatively weak interlayer van der Waals forces (Fig. 2g). To estimate the growth rate of F-Cu 2D layered crystals, we monitored an in situ real-time crystallization process using optical microscopy. Briefly, a quartz cuvette (10 mm) was filled with a freshly prepared alkaline solution of F, mixed with CuCl_2_ at a 2:1 molar ratio, and immediately capped to prevent evaporation or concentration changes. The crystallization process inside the cuvette was monitored under a light microscope at 2-s intervals (Supplementary Movie 1). In solution, small nucleation seeds appeared within a few seconds (~28 s) and laterally extended into large two-dimensional free-standing crystals (Fig. 2a, b). A selected single-crystal elongation growth was plotted as a function of time and indicated an estimated elongation rate of 0.5 nm/s at a linear regime (Fig. 2c and Supplementary Movie 2), revealing a spontaneous crystallization process. The corresponding snapshots of three consecutive images at different time points during the crystal growth are shown in Fig. 2c. These F-Cu crystals were further analyzed by high-resolution scanning electron microscopy (HRSEM) and atomic force microscopy (AFM). HRSEM micrographs showed laterally-extended rectangular 2D sheets with 10–500 μm lateral dimensions and layered hierarchy within each 2D crystal (Supplementary Fig. 1). AFM topographical measurements showed 260-nm-thick 2D crystals composed of several stacks of much thinner 10–20 nm nanolayers (Fig. 2h). Furthermore, owing to the week interlayer van der Waals interactions, the thicker sheets could be readily exfoliated into thinner 2D nanosheets with the assistance of external mechanical shear forces triggered by mild ultrasonication. The AFM topography images after ultrasonication treatment displayed ultrathin ∼1.8 nm nanosheets, each comprised of a small number of layers (Fig. 2i) inferred to be atomic monolayers based on the estimated thickness of ∼1.6 nm deduced from the X-ray crystal structure (Fig. 2g). In addition, the powder X-ray diffraction (PXRD) patterns of the F-Cu nanosheets and microcrystals were well-matched, revealing the intact nature of the highly crystalline 2D nanosheets (Fig. 2j). The UV-visible absorption spectrum of F-Cu nanosheets displayed a new broad 615 nm absorption band in the visible region assigned to the *d*-*d* transition specific to the Cu^2+^ complexes with tetragonal distortion owing to the Jahn–Teller effect (Supplementary Fig. 2). The Fourier-transform infrared spectrum (FTIR) of F-Cu nanosheets showed a new broad metal-oxygen stretching band at 555 cm^−1^ (ν_M-O_) and a significant red-shifted (Δν = 125 cm^−1^) amine absorption band, suggesting strong in-plane carboxylate- and amine-Cu^2+^ coordination modes (Supplementary Fig. 2). Energy dispersive X-ray (EDX) spectral analysis confirmed the presence of Cu atoms along with carbon (C) and oxygen (O) within the F-Cu nanosheets (Supplementary Fig. 2). Thermal gravimetric experiments exhibited relatively high thermal stability of the F-Cu nanosheets with two decomposition temperatures at 273 °C (68.8%) and 332 °C (14.3%) (Supplementary Fig. 2).

### Evaluation of the catalytic performance of F-Cu bionanozymes

**Fig. 3:**
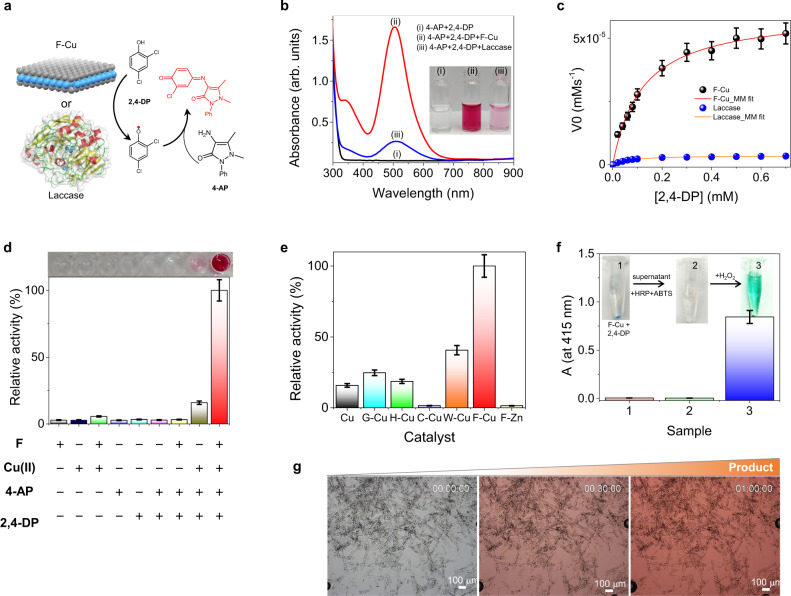
The catalytic activity of the F-Cu bionanozyme mimicking laccase. **a** Schematic illustration of the reaction utilizing 2,4-DP and 4-AP catalyzed by F-Cu nanosheets or laccase. **b** UV−vis absorption spectra of the two substrates (i) without any catalyst, and after their reaction in the presence of (ii) F-Cu and (iii) laccase. Reaction conditions: 0.6 mM 2,4-DP, 0.5 mM 4-AP, 5.04 × 10^−6^ mM (0.1 mg/mL) F-Cu, and 1.55 × 10^−3^ mM (0.1 mg/mL) laccase in PBS buffer (1X), pH 7.25 at 25 °C. Inset: Photographs of the corresponding solutions. **c** Initial rate (*V*_0_) of the reaction catalyzed by 0.1 mg/mL of F-Cu or laccase as a function of substrate concentration ([2,4-DP] = 0–0.7 mM, [4-AP] = 0.5 mM) (*n* = 3 independent experiments). **d** Relative catalytic activity in control experiments. No catalytic activity is observed when either F or Cu^2+^ is omitted from the system (*n* = 3 independent experiments). **e** Relative activity of several different amino acid-copper complexes (*n* = 3 independent experiments). **f** Control experiment testing the activity of the F-Cu bionanozyme and the supernatant that mimics the laccase. Photographs of (1) F-Cu reacted with 2,4-DP in PBS buffer (1X) (pH 7.25 at 25 °C) after centrifugation (12,000 rpm, 3 min), (2) the supernatant with ABTS and HRP added after centrifugation (12,000 rpm, 3 min), and (3) after adding H_2_O_2_ to (2). UV−vis absorbance at 415 nm of the corresponding samples (*n* = 3 independent experiments). **g** Optical microscopy snapshots at different intervals (0 min, 30 min, and 1 h) taken from the in situ reaction monitoring of 2,4-DP and 4-AP catalyzed by F-Cu crystals (Supplementary Movie 3), displaying the solution color change going from colorless (at 0 min) to red (after 30 min and 1 h). Error bars in all graphs represent standard deviations of three independent measurements.

2D layered nanomaterials have long been shown to be excellent materials of choice in the field of catalysis due to their unique structural and electronic properties originating from their ultrathin morphology and large surface area^38^. Therefore, the F-Cu crystals possess the advantages of easier and more rapid synthesis compared to natural enzymes and also meet the prerequisites for an efficient catalytic system (an ultrathin nanolayered structure accompanied by ample catalytically active redox sites), making them promising bionanozymes. Hence, the intrinsic catalytic activity of F-Cu nanosheets for mimicking the laccase function was first evaluated by performing the benchmarked oxidation reaction of 2,4-dichlorophenol (2,4-DP) together with 4-aminoantipyrine (4-AP)^39^. Here, 2,4-DP is the real substrate, and its radical product of 2,4-DP (generated by F-Cu bionanozyme- or laccase- catalyzed oxidation of 2,4-DP) reacts with 4-AP to produce a red-colored antipyrilquinoneimine dye with a characteristic broad absorbance centered at 510 nm (Fig. 3a, b). The 2,4-DP and 4-AP reaction mixture in the absence of a catalyst did not show any significant coloration or a product absorption peak. F-Cu nanosheets generated a more intensely colored product and a corresponding 510 nm absorption band compared to laccase (Fig. 3b and Supplementary Movie 4) at the same weight (0.1 mg/mL), suggesting that F-Cu possesses an outstanding laccase-mimicking catalytic activity. Further, the dependence of the initial rate of the reaction on the substrate concentration for F-Cu was consistent with its enhanced activity compared to the laccase enzyme (Fig. 3c). The typical Michaelis–Menten model fitting corroborated the enzyme-like catalytic characteristics of F-Cu nanosheets, with a maximum initial velocity of *V*_max_ = 6 × 10^−5^ mM/s and a Michaelis−Menten constant of *K*_M_ = 0.19 mM, while laccase has *V*_max_ = 3 × 10^−6^ mM/s and *K*_M_ = 0.06 mM. Furthermore, as shown in Table 1, the calculated turnover number (*k*_cat_ = 11.9 s^−1^) and catalytic efficiency (*k*_cat_/*K*_M_ = 62.65 mM^−1^ s^−1^) of the F-Cu bionanozyme are much higher than that of laccase (*k*_cat_ = 1.9 × 10^−3^ s^−1^; *k*_cat_/*K*_M_ = 0.03 mM^−1^ s^−1^). Moreover, F-Cu utilizes a single amino acid (F), making this system significantly less complex than the laccase enzyme comprised of a primary sequence of several hundred amino acids. Hence, in terms of molecular weight, F-Cu is the smallest known laccase mimicking bionanozyme and exhibits more than six orders greater catalytic efficiency per molecular weight ((*k*_cat_/*K*_M_)/M.Wt. = 160 × 10^−3^ (g l^–1^)^−1^ s^−1^) than the native laccase enzyme ((*k*_cat_/*K*_M_)/M.Wt) = 4.0 × 10^−7^ (g l^–1^)^−1^ s^−1^) (Table 1). In addition, when compared with previous laccase mimicking nanozymes, the F-Cu bionanozyme in this work displays the highest specific catalytic activity (Supplementary Table 2).

**Table 1:** Kinetic parameters of the F-Cu bionanozyme and laccase enzyme for oxidizing 2,4-DP at 25 °C.

Catalyst	#AA	M.Wt (Da)	[E] (mM)	*K*_M_ (mM)	*V*_max_ (mM s^−1^)	*k*_cat_ (s^−1^)	*k*_cat_/*K*_M_ (mM^−1^ s^−1^)	(*k*_cat_/*K*_M_)/M.Wt ((g l^−1^)^−1^ s^−1^)
F-Cu	1	392	5.04 ⋅ 10^−6^	0.19	6 ⋅ 10^−5^	11.9	62.65	160 × 10^−3^
Laccase	~521	~80,000	1.55 × 10^−3^	0.06	3 × 10^−6^	1.9 × 10^−3^	0.03	4.0 × 10^−7^

*#AA* number of amino acids, *M.Wt* molecular weight.

To further confirm the role of the F-Cu bionanozyme, control experiments were carried out using all possible combinations of catalyst building blocks. As shown in Fig. 3d, apart from F-Cu, Cu^2+^ alone showed only 16% activity with respect to F-Cu and produced a light-colored product. However, none of the other combinations showed any significant catalytic activity. These results indicate that the catalytic activity indeed depends on the F-Cu nanosheets rather than any non-assembled free F and Cu^2+^ ionic species. To better understand the catalysis mechanism, copper complexes of four different amino acids (simple amino acid glycine (G-Cu), aromatic amino acid tryptophan (W-Cu), and the amino acids present at the laccase active center: histidine (H-Cu) and cysteine (C-Cu)) were synthesized, and their catalytic activities were compared to that of the F-Cu bionanozyme (Fig. 3e). Among these, the F-Cu nanozyme displayed the highest activity (100%), followed by W-Cu (40%), G-Cu (25%), H-Cu (19%), Cu^2+^ ions (16%), and C-Cu (1.5%). Next, we replaced the divalent copper ions with zinc ions (Zn^2+^) by preparing an F-Zn complex and measuring its catalytic activity. However, F-Zn showed a negligible activity (1.4%, Fig. 3e), emphasizing the crucial role of Cu^2+^ in this reaction, similar to natural laccases. Therefore, the interaction between Cu^2+^ and F to form a highly crystalline 2D layered nanostructure is required for catalytic activity, whereas the carboxylate and amine functional groups in the amino acid skeleton support the coordination sites.

Many oxidases, such as glucose oxidase, accelerate a similar phenol oxidation reaction via catalytic reduction of O_2_ to H_2_O_2_. In contrast, laccase directly reduces molecular oxygen to water without any production or requirement of H_2_O_2_. To test whether H_2_O_2_ was generated during the reaction catalyzed by the F-Cu bionanozyme, a reaction mixture of 2,4-DP and F-Cu (4-AP was omitted to avoid interference of red color) was centrifuged (Fig. 3f). 2,2′-azino-bis(3-ethylbenzothiazoline-6-sulfonic acid (ABTS) and horseradish peroxidase (HRP) were added to the supernatant, yet no variation of color or absorption was observed (inset of Fig. 3f), indicating the absence of H_2_O_2_. However, under similar conditions, the addition of H_2_O_2_ resulted in a green-colored solution with an intense absorbance at 415 nm, characteristic of the ABTS oxidized product. These results further confirm that the F-Cu bionanozyme is indeed a laccase mimic rather than any other oxidase that generates an H_2_O_2_ by-product. Since the laccase activity depends on molecular oxygen, we further tested the oxygen dependency of the F-Cu catalytic activity by conducting the reaction in the presence (open to air) and absence (nitrogen gas bubbled solution) of an oxygen-containing environment (Supplementary Fig. 5). Interestingly, the catalytic reaction rate in the presence of atmospheric oxygen was 2.5 times higher than in the absence of oxygen. This confirms the oxygen dependency of F-Cu catalyzed oxidation of phenols, similar to the natural laccase enzyme. The oxidation state of Cu^2+^ within the F-Cu bionanozyme during the reaction was probed through electron paramagnetic resonance (EPR) spectroscopy (Supplementary Fig. 5). The F-Cu bionanozyme alone displayed an intense EPR signal at 3291 G characteristic of Cu^2+^. However, the addition of 2,4-DP to the F-Cu reaction mixture resulted in a diminished EPR signal, suggesting that the reduction of Cu^2+^ to Cu^1+^ occurred, in line with the anticipated laccase mimicking redox catalytic mechanism. To visualize the progress of the F-Cu bionanozyme catalytic oxidation of 2,4-DP, in situ microscopy (Supplementary Movie S3) experiments were performed for 1 h at 15-s intervals (Fig. 3g). The colorless reaction mixture turned into a strong red color on the surface of the 2D F-Cu crystals without affecting their morphology, providing further evidence of the catalytic function of the F-Cu 2D crystals. In addition, filtration experiments (Supplementary Fig. 6) further suggest that the catalytic function is exclusively from F-Cu single crystals and excludes any possible release of Cu^2+^ ions and contribution from dissolved or non-assembled structures.

### Catalytic stability, recyclability, and substrate universality of F-Cu bionanozymes

**Fig. 4:**
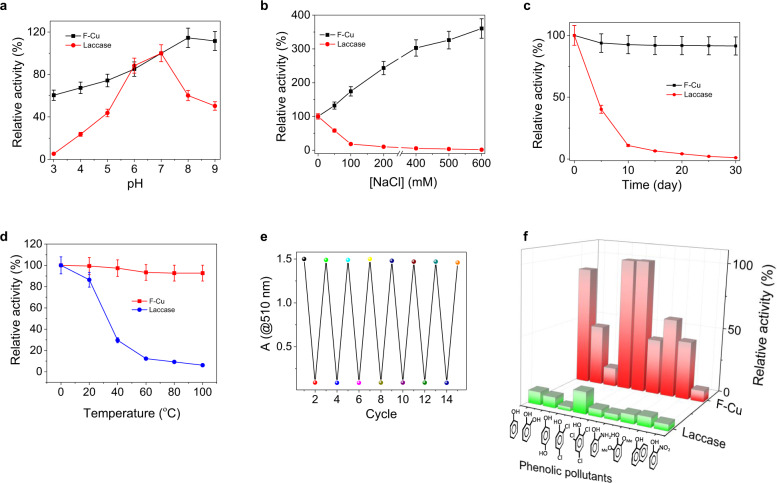
Catalytic stability, reusability, and substrate universality of F-Cu bionanozyme. **a**–**d** Relative activity of F-Cu and laccase under various **a** pH values (*n* = 3 independent experiments), **b** ionic strength (NaCl concentration, *n* = 3 independent experiments.), **c** storage time (*n* = 3 independent experiments), and **d** incubation temperature (*n* = 3 independent experiments). **e** Relative activity during the F-Cu recycling process (conditions for the recycling experiments: 0.6 mM 2,4- DP, 0.5 mM 4-AP, 5.04 × 10^−6^ mM (0.1 mg/mL) (F-Cu, pH 7.25, 25 °C, 1 h)). **f** comparison of relative catalytic conversion activity of the F-Cu bionanozyme and laccase based on the same weight (0.1 mg/mL) for various toxic phenolic pollutants (left to right: phenol, Catechol, Hydroquinone, 2,4- DP, 2,4,6-trichlorophenol, 2-Aminophenol, 2,6-Dimethoxyphenol, 2-Naphthol, and 2-Nitrophenol). Error bars in all graphs represent standard deviations of three independent measurements.

To demonstrate its practical utility, we investigated the relative stability of the F-Cu bionanozyme compared to laccase in various extreme experimental conditions, including pH, ionic strength, storage time, and temperature. Specifically, the catalytic assay was performed in PBS buffer (1X, 25 °C) at different pH values ranging from pH 3.0 to 9.0. As shown in Fig. 4a, laccase lost its catalytic activity after incubation in a strongly acidic pH 3.0 (5%) and basic pH 9.0 (48%), while the F-Cu bionanozyme maintained at least 60% of its catalytic activity at pH 3.0 and gradually increased at pH 4.0-9.0 (65–120%). The influence of ionic strength on catalytic activity was tested at different NaCl concentrations ([NaCl] = 0 to 600 mM). The increase in ionic strength induced a drastic decrease in laccase activity (from 100 to 1.4%) due to the salting-out effect and chloride ion inactivation (Fig. 4b). In contrast, F-Cu gradually increased its activity (100 to 360%) with elevated NaCl concentration. This result prompted us to test the catalysis in actual water samples, including tap water, river water, and seawater (Supplementary Fig. 4). F-Cu and laccase showed similar relative activity in both tap and river water. However, laccase completely lost its activity in seawater, while F-Cu retained 76% of its catalytic activity. Therefore, the F-Cu bionanozyme is robust enough for on-field phenol oxidation in water samples highly relevant for public health. Next, the storage stability was estimated by loading the catalyst in PBS buffer (1X, pH 7.4) at 25 °C and assayed every 5 days for 1 month. As shown in Fig. 4c, the relative activity of laccase gradually decreased over storage time and became completely inactive on the 10th day. However, the F-Cu bionanozyme retained almost 90% of its activity after 30 days, as well as remained 95% active after 210 days in the air at room temperature, indicating the excellent stability of the F-Cu bionanozyme during storage in either water or air. Further, thermal stability was evaluated by incubating F-Cu and laccase at different temperatures ranging from 0 to 100 °C for 1 h, followed by a catalytic activity assay at room temperature (Fig. 4d). Due to thermal denaturation, the relative activity of laccase decreased gradually and was completely lost at 60 °C. On the other hand, F-Cu bionanozymes exhibited good thermal stability by retaining 92% activity even after incubation at 100 °C. To demonstrate the recyclability of the F-Cu bionanozymes, both 2,4-DP and 4-AP were mixed with F-Cu in PBS buffer (1X, at 25 °C) for each cycle. After each reaction cycle, the F-Cu bionanozyme was collected by centrifugation (12,000 rpm, 3 min), washed with double-distilled water, and reused for the next reaction cycle (Fig. 4e). Remarkably, the F-Cu bionanozyme retained more than 80% of its relative activity even after fifteen cycles, while laccase could not be recycled. These results suggest that the F-Cu bionanozyme shows higher catalytic stability and recyclability compared to natural laccase. To assess the universality of the substrate, F-Cu and laccase were mixed with various toxic phenolic contaminants, and the catalytic oxidation activity was assayed (Fig. 4f). Interestingly, the F-Cu bionanozyme could catalyze the oxidation of all phenolic contaminants tested and also showed higher catalytic activities compared to laccase, demonstrating the favorable substrate universality of the F-Cu bionanozyme. Importantly, F-Cu showed a higher conversion rate of chlorophenols (2,4-DP and 2,4,6-trichlorophenol) which are listed by the US Environmental Protection Agency (EPA) as priority environmental pollutants^40^.

### Reaction pathway for F-Cu catalytic oxidative coupling of phenolic pollutants

**Fig. 5:**
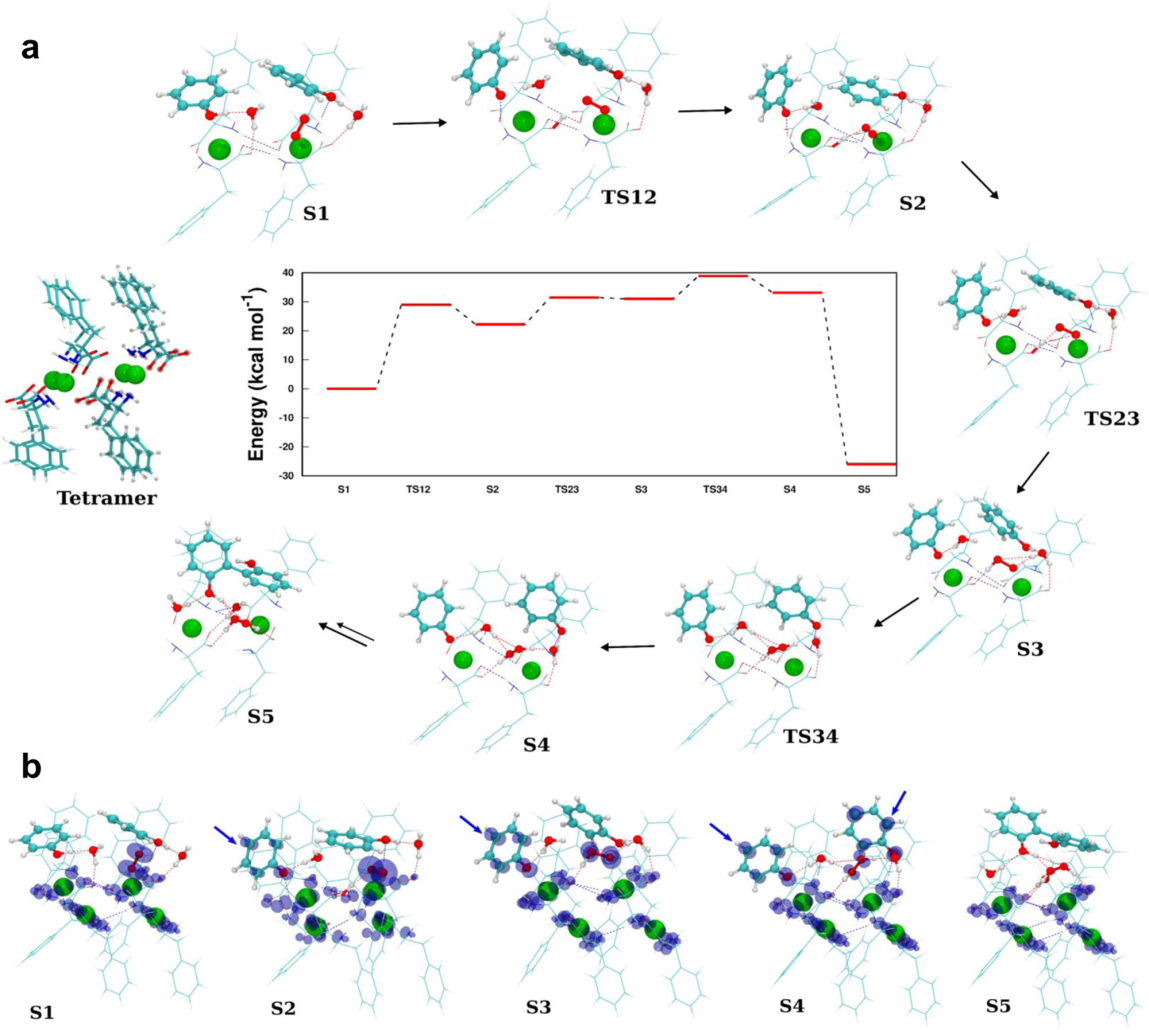
Reaction pathway for F-Cu catalytic oxidative coupling of phenolic pollutants. **a** The tetramer model of F-Cu used in the DFT calculations is shown on the left. The atoms displayed as transparent beads in the tetramer were held fixed throughout the calculations to emulate periodic crystal effects. Relative energies of steps 1 through 5, with transition state energies scaled with respect to step 1, are shown in the central part of the panel. The optimized structures of all steps are also shown, with fixed atoms not shown for clarity. **b** Spin-densities of steps 1 through 5. Spin-densities on the phenyl rings of the phenoxyl radicals are indicated by the blue arrows.

To understand the energetics of phenoxyl radical formation, we carried out first-principle spin-polarized density functional theory (DFT) calculations on a cluster model of F-Cu comprised of four Cu(F)_2_ units as shown in Fig. 5a (left). Our cluster model resembles an edge of the F-Cu crystal (100) surface. To obtain the energetics of the entire reaction, we added one oxygen, two water, and two phenol molecules to this tetramer model of F-Cu. Furthermore, to mimic periodic crystal effects, we fixed the positions of two of the four Cu(F)_2_ units (located on the right side of the tetramer, represented with both sticks and transparent beads).

The entire reaction starts with a phenol molecule approaching the active copper site (step 1). In this step, the spin-density is primarily located near the four copper sites (see S1 in Fig. 5b) and on the oxygen molecule. Next, there is a transfer of a hydrogen atom from the phenol molecule to one of the –COO groups of the F moiety (step 2), with an energy barrier of ~29 kcal/mol. Step 2 is ~22 kcal/mol higher in energy than step 1, and due to the transfer of a hydrogen atom from the phenol, a phenoxyl radical is formed in this step. We confirmed the formation of a phenoxyl radical through spin-density plots (Fig. 5b), where we observed an accumulation of spin-density on the phenyl moiety of the phenol in step 2, which was absent in step 1. In the next step, the hydrogen atom is further transferred from the –COO group to the oxygen molecule, with an energy barrier of ~9.5 kcal/mol. In the experimental studies, molecular oxygen is readily available in the solvent (water) as dissolved oxygen. Until step 3, the reaction is energetically uphill; however, once this phenoxyl radical diffuses from the active copper site, it interacts with other phenoxyl radicals forming the respective polymers, resulting in an overall exothermic reaction. To simulate this downhill process, we studied the formation of a dimer (2,2′-biphenol) from two phenols. To reduce the computational burden associated with the simulation of this large system, we assumed that both phenols interact with the adjacent copper sites. In step 4, we observed the formation of two phenoxyl radicals on the adjacent copper sites, where the oxygen molecule receives protons from both phenols. The spin-density plots confirm the radical nature of the phenoxyl moieties. It is interesting to note that this configuration containing two phenoxyl radicals on adjacent copper sites is only slightly higher in energy (~2 kcal/mol) compared to step 3. Finally, the two radicals combine with each other to form the polymerized product that is ~55 kcal/mol lower in energy compared to step 3 or step 4.

Overall, our computational studies strongly suggest that the transfer of the H atom to the –COO group is one of the crucial steps in the catalytic oxidation of phenols. This is further supported by in situ Raman measurements (Supplementary Fig. 7) conducted on the F-Cu complex before and after the addition of 2,4-DP (significant changes observed in the vibrational modes of the carboxylate moiety indicates the weakening of Cu^2+^ binding with the carboxylate ion, possibly due to the transfer of an H atom to carboxylate (–COO) groups of F-Cu). Furthermore, since the phenyl moiety retained its spin-density in all the other intermediate steps, the H atom transfer continues in all these steps. Finally, although there are several endothermic intermediate reactions prior to the polymerization of the phenoxyl radicals, since the polymerization step itself is extremely exothermic, the entire reaction is thermodynamically feasible, as observed in the experiments.

### Detection of catecholamine neurotransmitter using the F-Cu bionanozyme

**Fig. 6:**
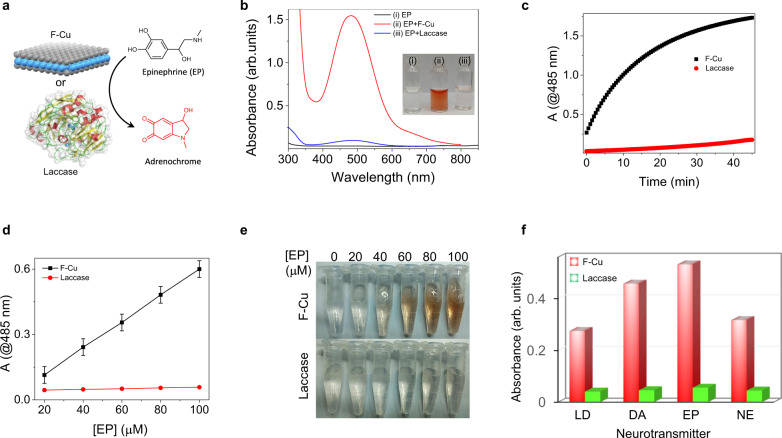
Colorimetric detection of catecholamine neurotransmitters. **a** Schematic reaction of colorless epinephrine (EP) oxidation into the chromogenic product catalyzed by F-Cu or laccase. **b** UV−vis spectra of (i) EP as well as its oxidation product catalyzed by (ii) F-Cu and (iii) laccase. **c** Comparison of the progress of epinephrine oxidation catalyzed by F-Cu and laccase. **d** Linear relationships between the absorbance at 485 nm and the concentration of EP in the presence of F-Cu and laccase (*n* = 3 independent experiments). Error bars represent standard deviations of three independent measurements. **e** Photographs of visible detection of different concentrations (5−30 μg/mL) of EP. **f** Comparison of the relative activity of F-Cu and laccase catalyzing the oxidation of four different catecholamine neurotransmitters substrates, L-DOPA (LD), dopamine (DA), epinephrine (EP), and norepinephrine (NE).

To further broaden the scope of the F-Cu bionanozyme, we examined the catalytic oxidation of catecholamines, which are biologically important phenolic neurotransmitters (dopamine (DA), epinephrine (EP), norepinephrine (NE), and l-DOPA (LD)). Catecholamines play a vital role in various functions of the cardiovascular, nervous, and endocrine systems^41^. Therefore, any disruption in their concentration levels may induce diverse diseases, including Parkinson’s disease, schizophrenia, and even tumors such as paraganglioma and pheochromocytoma. On the other hand, catecholamines are extensively used as drugs for emergency heart disease, anaphylactic shock, and bronchial asthma. Hence, the detection and quantitative analysis of catecholamines are essential for disease diagnosis and pharmaceutical assessments. Interestingly, simple mixing of F-Cu or laccase with EP resulted in a gradual color change in the solution from colorless to intense red, indicating the formation of an oxidized product (Fig. 6a). This was further evident from the appearance of a new absorption band centered at 485 nm, characteristic of an adrenochrome product (Fig. 6b). In contrast, in the absence of a catalyst (F-Cu or laccase), EP did not produce a significant color change. The F-Cu bionanozyme-catalyzed product absorption and solution coloration were much higher than those observed for laccase. The catalytic course of F-Cu and laccase monitored at 485 nm over 30 min is shown in Fig. 6c. Remarkably, in the first 10 min, the reaction kinetics with the F-Cu bionanozyme is 20 times faster than that of laccase. Furthermore, the calculated kinetics parameter *V*_max_ of the F-Cu bionanozyme is ∼43 times higher than laccase. The catalytic colored oxidized product could be used as a method for the detection and quantification of EP. To test this option, we monitored the absorbance of the product at 485 nm as a function of EP concentration in the presence of the same weight (0.1 mg/mL) of the F-Cu bionanozyme and laccase (Fig. 6d, e). Both F-Cu and laccase displayed a linear relationship between the product absorbance and the concentration of EP between 20 to 100 μM. The obtained limit of detection (LOD) of EP is 150 nM in the presence of the F-Cu bionanozyme, compared to 5 μM in the presence of laccase. Accordingly, F-Cu is ∼36 times more sensitive than laccase. Therefore, the method utilizing the F-Cu bionanozyme is much more simple, ultrasensitive, and allows the same conversion rate that is ∼5400 times more cost-effective compared to the natural enzyme laccase. Next, we analyzed the catalytic oxidation of other catecholamines (DA, NE, and LD) in the presence of the F-Cu bionanozyme and laccase. As shown in Fig. 6f, the F-Cu bionanozyme could catalyze the oxidation of all types of catecholamine molecules more effectively than laccase. Therefore, based on the F-Cu bionanozyme catalytic oxidation reactions, a rapid, economical, and ultrasensitive methodology has been developed for the colorimetric determination of catecholamine neurotransmitters.

## Discussion

In conclusion, we describe an important step towards developing eco-friendly, cost-effective, efficient, stable, and reusable minimalist bionanozymes for environmental remediation. Unlike natural enzymes consisting of several hundred amino acid sequences, the designed F-Cu bionanozyme utilizes only a single amino acid, F, which spontaneously coordinates with Cu^2+^ ions to produce catalytically ideal hierarchical 2D layered van der Waals crystals. This simple supramolecular approach provides an attractive alternative to the expensive, time-consuming, and inaccessible chemical synthesis and purification processes. The F-Cu bionanozyme reported here uses 2D orderly-spaced Cu^2+^/Cu^1+^ sites for mimicking the biocatalytic action of the natural enzyme laccase, effectively oxidizes a broad range of widespread environmentally toxic phenolic contaminants, and serves as an ultrasensitive tool to detect biologically important neurotransmitters. The ultrathin 2D layered structure of the F-Cu bionanozyme provides a high surface area and a larger number of accessible active sites, thus showing more than four orders of higher catalytic efficiency (*k*_cat_/*K*_M_ = 62.65 mM^−1^ s^−1^) than the laccase enzyme (*k*_cat_/*K*_M_ = 0.03 mM^−1^ s^−1^). Compared to the natural laccase enzyme, the developed F-Cu bionanozyme displays excellent stability under extreme conditions, recyclability, and wide substrate universality, thus having great potential for environmental remediation, industrial, and healthcare applications. The ease with which we were able to develop the robust single amino acid bionanozyme might provide an important missing link in the quest for prebiotic catalysts during the evolution of enzymes. Moreover, given the rich structural diversity and versatile metal coordination characteristics of amino acids, this report provides a rational path towards the de novo design of next-generation minimalistic bionanozymes for energy, environment, and healthcare applications.

## Methods

### Materials

l-phenylalanine (F), glycine (G), l-Cysteine (C), l-Histidine (H) copper (II) chloride dihydrate (CuCl_2_), Sodium hydroxide (NaOH), 2,4-dichlorophenol (2,4-DP), 4-aminoantipyrine (4-AP), 2,2′-azino-bis(3-ethylbenzothiazoline-6-sulfonic acid (ABTS), peroxidase from horseradish (HRP), Hydrogen peroxide solution (H_2_O_2_), *N*-methyl-2-pyrrolidone (NMP), phenol, catechol, hydroquinone, 2-aminophenol, 2,6-dimethoxyphenol, 2-naphthol, 2-nitrophenol, 2,4,6-trichlorophenol, epinephrine (EP), norepinephrine (NP), dopamine, l-DOPA, sodium chloride (NaCl), potassium chloride (KCl), disodium phosphate (Na_2_HPO_4_), and potassium phosphate monobasic (KH_2_PO_4_) were purchased from Sigma-Aldrich (Rehovot, Israel). All materials were used as received. Milli-Q water was used to prepare all the buffers and solutions.

### Preparation of F-Cu single crystals

One equivalent of the CuCl_2_ (5 mM) aqueous solution was slowly added to two equivalents of an l-phenylalanine (10 mM) alkaline solution (containing NaOH (10 mM)) under heating (60 °C). Thin plate-like crystals were spontaneously formed at the liquid–air interface. The crystals were collected by filtration and washed with an excess of double-distilled water and ethanol. The in situ crystal growth was recorded using optical microscopy at 14-s intervals.

### Single-crystal X-ray data collection and processing

Blue plate-like crystals suitable for diffraction were coated with Paratone oil (Hampton Research) and mounted on MiTeGen loops and flash-frozen in liquid nitrogen. All X-ray diffraction measurements were carried out at 100 K. Diffraction measurements for F-Cu crystals were performed at the ESRF synchrotron, station ID23-1. Data were collected and processed using MXCube and the automated XDS pipeline. The structure was solved by direct methods using SHELXT-2013 or SHELXT 2016/4. The structure was refined by full-matrix least-squares against F2 with SHELXL 2016/4. The crystallographic data are given in Table S1. The structure was illustrated using Mercury 3.9 (Cambridge Crystallographic Data Centre, Cambridge, UK).

### Powder X-ray diffraction

The F-Cu nanosheets were deposited on a quartz zero-background sample holder, and powder XRD was performed on a Bruker D8 Advance X-ray powder diffractometer (Bruker) at room temperature between 5 and 45° 2θ. The background was subtracted to extract the diffraction peaks.

### FTIR spectroscopy

The F-Cu nanosheets were deposited on disposable KBr infrared sample cards (Sigma-Aldrich) and subsequently dried under vacuum. The spectra were collected from 4000 to 400 cm^−1^ at room temperature using a nitrogen-purged Nicolet Nexus 470 FTIR spectrometer (Nicolet) equipped with a deuterated triglycine sulfate detector. One hundred and twenty-eight scans were collected with a spectral resolution of 4 cm^−1^. The background was subtracted from a control KBr spectrum.

### High-resolution scanning electron microscopy (HRSEM)

The as-prepared F-Cu crystals were deposited on a clean microscope glass coverslip and coated with Cr. The images were recorded under a JSM-6700 field emission scanning electron microscope (JEOL, Tokyo, Japan) operated at 10 kV.

### Atomic force microscopy (AFM)

The F-Cu crystals before and after ultrasonication treatment (for 10 min) were deposited onto a clean microscope glass coverslip. The samples were characterized using the AIST-NT Smart AFM system in non-contact (tapping) mode, 100-mm-long silicon nitride cantilevers (OMCL-RC800PSA-W, Olympus) with a resonance frequency of 70 kHz. The images were analyzed and visualized using WSxM imaging software (Nanotec Electronica S.L).

### Thermal gravimetric analysis (TGA)

TGA experiments were performed on a TA Instruments (USA) module SDT 2950 at a temperature range between 40 and 510 °C, with a heating rate of 10 °C min^−1^ under dry ultrahigh-purity argon atmosphere.

### Electron paramagnetic resonance (EPR)

EPR spectra were acquired in Wilmad tubes at 10 K using a Bruker EMX EPR spectrometer equipped with a cryostat. The freshly prepared F-Cu bionanozyme in NMP (30 μL from 1 mg/mL) was added to a 240 μL PBS buffer (1X, pH 7.25) and transferred into an EPR tube flash-frozen in liquid nitrogen. The final pH of the sample was 7.6 as measured by a Spintrode electrode (Hamilton). In total, 30 µL of DMP solution in i-PrOH (70 mM) was added directly to the EPR tube, which was inverted five times and frozen in liquid nitrogen. The data were plotted using Origin.

### Raman spectroscopy

Raman spectroscopic measurements were performed on the samples using an Xplora Raman spectrometer (HORIBA Jobin-Yvon) equipped with a monochromatic laser of 532 nm wavelength. The spectra were collected at different times from the F-Cu substrate before and after adding a 2,4-DP solution (1 mM). The laser power at the sample surface was maintained at 1 mW (typical power density ~3.18 × 10^4^ W/cm^2^). The typical data acquisition time used was 30 s. The collected spectra were normalized with respect to the intensity of breathing mode of the Benzene ring (~1000 cm^−1^) and plotted using the Origin program.

### Catalytic assay and kinetic parameters determination

The F-Cu bionanozyme catalytic performance was measured by the chromogenic reaction of phenolic compounds with 4-AP using a 96-well UV-Star UV transparent flat bottom plate (Greiner BioOne, Frickenhausen, Germany). First, 4-AP (30 μL from 5 mM i-PrOH solution) and 2,4-DP (30 μL from 6 mM i-PrOH solution) solutions were mixed with PBS buffer (1X, pH 7.4, 210 μL). Next, F-Cu bionanozyme in NMP or laccase enzyme (30 μL from 1 mg/mL) was added. The reaction progress was monitored at 510 nm using a Biotek Synergy HT plate reader (Biotek, Winooski, VT, USA) at 22 °C and continuous stirring mode. To obtain the initial reaction rate (V_0_) various concentrations of 2,4-DP (0.02, 0.04, 0.06, 0.08, 0.1, 0.2, 0.3 0.4, 0.5, 0.6, and 0.7 mM) were respectively reacted with 0.5 mM of 4-AP, catalyzed by 0.1 mg/mL of F-Cu bionanozyme or laccase. The average of the *V*_0_ values was plotted as a function of substrate concentration and fit to the Michaelis–Menten equation (*V*_0_ = *V*_max_ [*S*]/(*K*_M_ + [*S*]) to obtain the kinetic parameters *V*_max_ and *K*_M_. In addition, we used the reported method to calculate *K*_cat_ for both laccase and F-Cu bionanozyme^42,43^. All measurements were replicated at least three times and averaged for accuracy. The other phenolic substrates (phenol, Catechol, Hydroquinone, 2-Aminophenol, 2,6-Dimethoxyphenol, 2-Naphthol, 2-Nitrophenol, and 2,4,6-trichlorophenol) at 0.6 mM were reacted with 0.5 mM 4-AP in PBS buffer (1X, pH 7.4, 210 μL) containing 0.1 mg/mL of F-Cu bionanozyme. The in situ experiments were done under an optical microscope by mixing 0.5 mM of 2,4-DP and 4-AP with F-Cu crystals (0.1 mg/ml) PBS buffer (1X, pH 7.4, 210 μL) solution. The reaction progress was recorded at 15-s intervals.

### Assessment of catalytic stability comparison

To probe the effect of pH, an F-Cu bionanozyme or laccase catalytic assay was performed similarly but at different pH (3.0−9.0) PBS buffer (1X) solutions. The relative activity was compared with that of pH 7. Different concentrations of NaCl (0, 50, 100, 200, 400, 500, and 600 mM) were added into the reaction mixture to measure the effect of ionic strength on the catalytic activity, and the relative activity was compared with that of 0 mM NaCl. To assess the practical utility of our enzymes, the F-Cu bionanozyme and laccase catalytic assays were measured in real water (instead of PBS buffer), including tap water, river water, and seawater as described above. The long-term storage stability was measured every 5 days for the residual activity of F-Cu bionanozyme or laccase enzyme dispersed in Milli-Q water stored at room temperature. The activity on the first day was taken as a reference. The effect of temperature was measured by incubating the F-Cu bionanozyme and laccase enzyme separately at 0, 20, 40, 60, 80, and 100 °C for 30 min, and their catalytic activity was subsequently assayed. The activity at 0 °C was taken as a reference. To evaluate the recyclability of F-Cu bionanozyme, the solutions of 2,4-DP (0.6 mM), 4-AP (0.5 mM), and F-Cu (0.1 mg/mL) were mixed in PBS buffer (1X, pH = 7.4,) at 25 °C, and the reaction was assayed for 1 h per cycle. After each reaction cycle, the F-Cu and bionanozyme were collected by centrifugation (12,000 rpm, 3 min), washed with Milli-Q water three times, and reused for the next reaction cycle.

### Reaction with epinephrine

The F-Cu bionanozyme or laccase enzyme (30 μL from 1 mg/mL solution) and epinephrine (30 μL from 5 mM *i-*PrOH solution,) were mixed with PBS buffer (1X, pH 7.4, 240 μL) in a 96-well UV-Star UV transparent flat bottom plate (Greiner BioOne, Frickenhausen, Germany). The reaction progress was monitored at 485 nm using a Biotek Synergy HT plate reader (Biotek, Winooski, VT, USA) at 22 °C and continuous stirring mode. To evaluate the detection limit of epinephrine, different concentrations of epinephrine (0, 20, 40, 60, 80, and 100 μM) were mixed with 0.1 mg/mL of F-Cu bionanozyme or laccase in the PBS buffer for 1 h at 25 °C before the 485 nm absorbance measurement. The detection limit was calculated by 3σ/b, where σ is the standard deviation of the blank signals, and b is the slope of the regression line.

### Computational details

Spin-polarized density functional theory (DFT) calculations were carried out using the PBE exchange-correlation functional and numerical orbital basis-sets as implemented in the all-electron code FHI-aims. Dispersion interactions were included using the Tkatchenko and Scheffler scheme. For each geometry, different spin-configurations were considered, and the configuration with the lowest energy was reported (a heptet configuration was used for steps 1 through 4, and a quintet was used for step 5. For step 3, the triplet and heptet configurations were found to be degenerate). In all calculations, we used a convergence criterion of 10^−5^ for the electron density and 10^−2^ eV Å^−1^ for the structural relaxation. Throughout these calculations, we used the “tight” settings for all the atomic species as available in the FHI-aims software package (the difference in the energies predicted using the “light” and “tight” settings were found to be less than 1 kcal/mol). For the climbing image calculations, we used “climb_mode = 2”, which corresponds to the optimization of both the climbing image as well as the two images close to it, and a convergence criterion of 0.05 eV Å^−1^ was utilized while evaluating the forces.

### Reporting summary

Further information on research design is available in the Nature Research Reporting Summary linked to this article.

## Supplementary information

Supplementary information

Description of Additional Supplementary Files

Supplementary Movie 1. In-situ monitoring of the F-Cu crystallization process under an optical microscope

Supplementary Movie 2. In-situ optical microscopy observation of phenylalanine-coordinated Copper ions (F-Cu) crystallization kinetics

Supplementary Movie 3. In-situ reaction monitoring of 2,4-DP and 4-AP catalyzed by F-Cu crystals

Supplementary Movie 4. Real-time monitoring of 2,4-DP and 4-AP oxidation in the presence and absence of the F-Cu catalyst

## Data Availability

Supplementary information and Supplementary movies are available in this article. The X-ray crystallographic coordinates for the structure reported in this study have been deposited in the Cambridge Crystallographic Data Centre (CCDC) database under accession code 1871975.
