# Supplementary material for: Single amino acid bionanozyme for environmental remediation
Source: Nat Commun. 2022 Mar 21;13:1505. doi: 10.1038/s41467-022-28942-0 (PMC8938493; doi:10.1038/s41467-022-28942-0)
Supplement: Supplementary file 1 — Supplementary information [file 41467_2022_28942_MOESM1_ESM.pdf]

SUPPLEMENTARY INFORMATION

Single amino acid bionanozyme for environmental remediation

Pandeeswar Makam[1]*, Sharma S. R. K. C. Yamijala[2,3,4], Venkata S. Bhadram[5], Linda J. W. Shimon[6], Bryan M. Wong[2]*, and Ehud Gazit[7,8]*

Affiliations:

[1] Department of Chemistry, Indian Institute of Technology (BHU), Varanasi, UP-221005, India.

[2] Department of Chemical & Environmental Engineering and Materials Science & Engineering Program, University of California-Riverside, Riverside, CA, 92521, USA.

[3] Department of Chemistry, Indian Institute of Technology Madras, Chennai - 600036, India.
[4] Center for Atomistic Modelling and Materials Design, IIT Madras, Chennai - 600036, India

[5] IMPMC, Sorbonne Université, CNRS, MNHN, 4, place Jussieu, 75005 Paris, France

Current address: Division of Sciences, Krea University, Sri City - 517646, India

[6] Chemical Research Support, The Weizmann Institute of Science, 7610001 Rehovot, Israel.

[7] The Shmunis School of Biomedicine and Cancer Research, Tel Aviv University, Tel Aviv 6997801, Israel.

[8] Department of Materials Science and Engineering, Iby and Aladar Fleischman Faculty of Engineering, Tel Aviv University, Tel Aviv 6997801, Israel.

*Correspondence: pandeeswar.chy@itbhu.ac.in, bryan.wong@ucr.edu, and ehudg@post.tau.ac.il

Supplementary Table 1. Crystallographic Data collection and refinement statistics of F-Cu

Compound	F-Cu
CCDC	1871975
Diffractometer	ESRF ID23-1
Empirical formula	$C_{18}H_{20}CuN_2O_4$
Formula weight (g/mol)	391.90
Crystal description	Blue plate
Temperature (K)	100
Wavelength (Å)	0.70
Crystal system	Monoclinic
Space group	$P2_1$
a, b, c (Å)	9.4800(19), 5.1500(10), 16.670(3)
α, β, γ (°)	90, 98.93(3), 90
Volume (Å^3)	804.0(3)
Z	2
Density calculated (mg/m^3)	1.619
Absorption coefficient (mm^{-1})	1.385
F(000)	406
Crystal size (mm)	0.15x0.10x0.01
Theta range for data collection (°)	1.218 to 31.926
Reflections collected (unique)	9071(5472)
R int	0.0435
Completeness (%)	95.3
Data\restraints\ parameters	5472/1/242
Goodness-of-fit on F^2	1.105
Final R [I>2σ(I)]	R1=0.0462, wR2=0.1234
R (all data)	R1=0.0475, wR2=0.1256
Largest diff. peak and hole (e$^.$ Å^{-3})	1.416 and -0.548

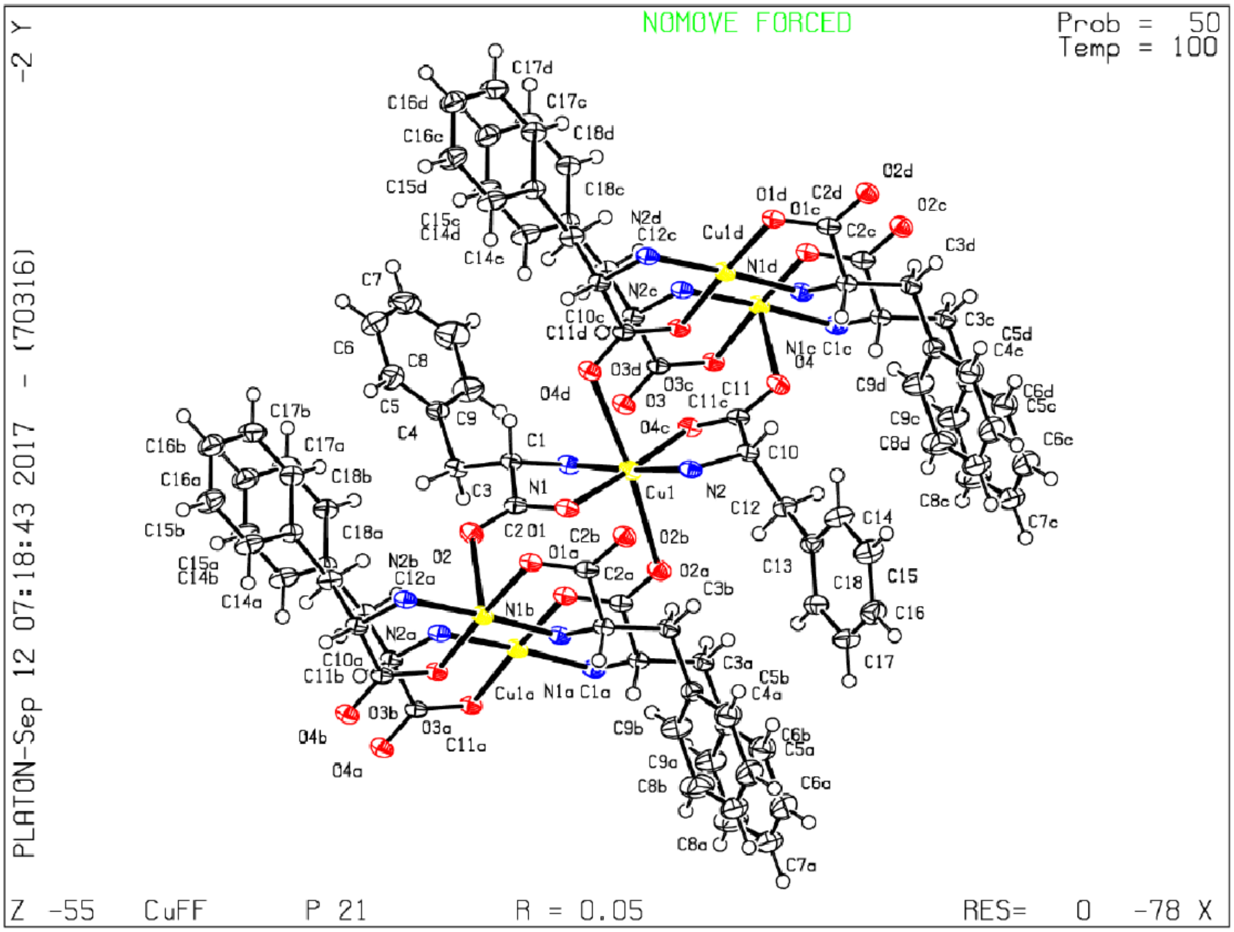

ORTEP diagram of the F-Cu crystal structure

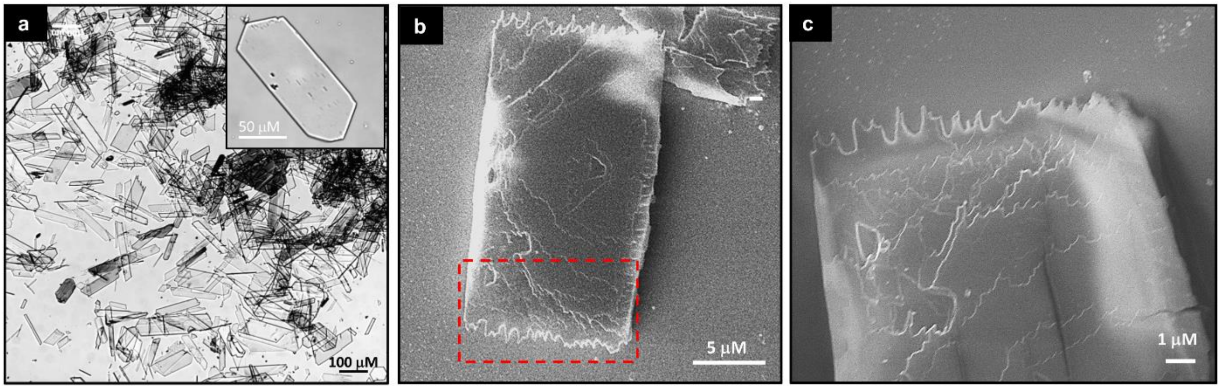

Supplementary Fig. 1. Microscopic images of F-Cu crystals. a, Optical microscope images. The inset depicts the enlarged single crystal. **b** and **c**, HRSEM images. Each experiment was repeated three times independently with similar results.

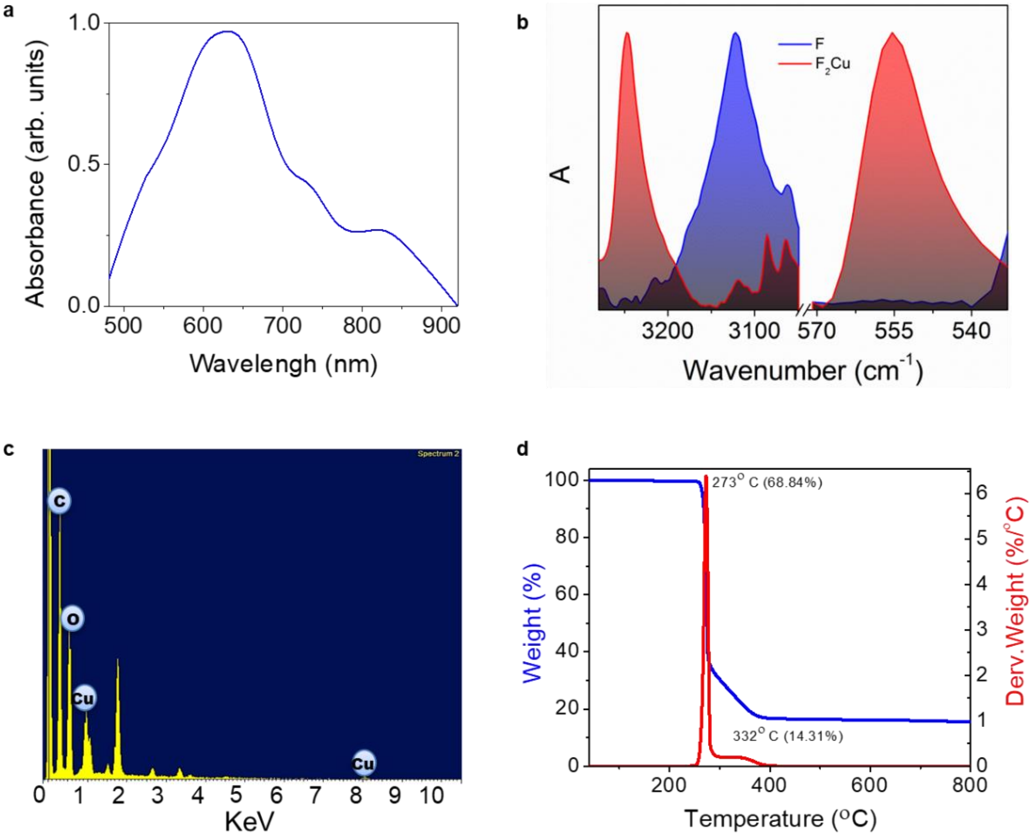

Supplementary Fig. 2. Spectroscopic characterization of the F-Cu bionanozyme. a, UV-vis absorption spectra. **b**, FTIR spectra (3270–3040 ($\nu_{N\text{-}H}$) and 580–540 cm^{-1} ($\nu_{M\text{-}O}$) region of phenylalanine (F) and F-Cu. **c**, EDS spectra. **d**, TGA of F-Cu bionanozyme (blue curve) revealing the high thermal stability (≤ 273 °C). The weight losses are depicted by the first derivative curve (red).

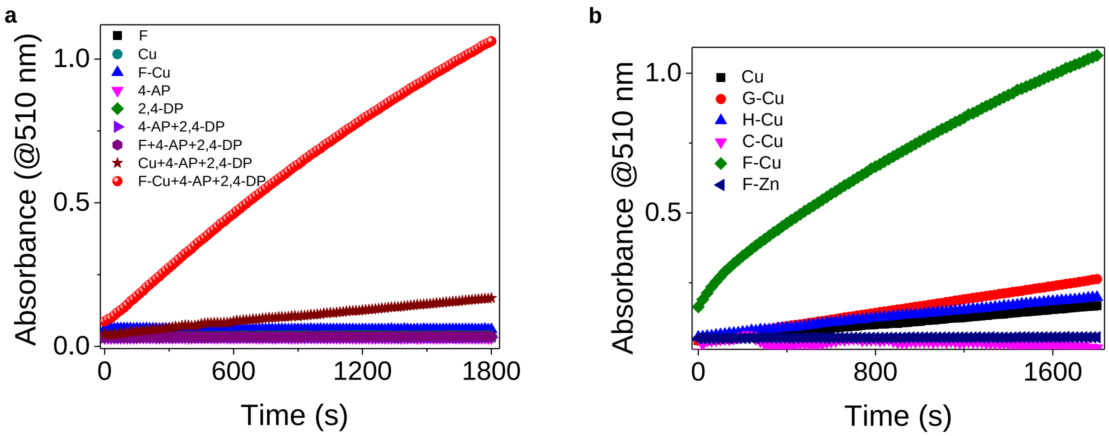

Supplementary Fig. 3. Catalytic activity in control experiments, a, Reaction progress in the presence of all the control building blocks of the F-Cu bionanozyme. Only the F-Cu bionanozyme can catalyze the oxidation of 2,4-DP into a colored product with an absorbance at 510 nm. No significant oxidation was observed when omitting either the phenylalanine (F) molecule or Cu from the catalytic system. **b**, Reaction progress in the presence of different copper complexes of amino acids (glycine (G-Cu), histidine (H-Cu), cysteine (C-Cu), and phenylalanine (F-Cu)) and the phenylalanine-zinc (F-Zn) complex.

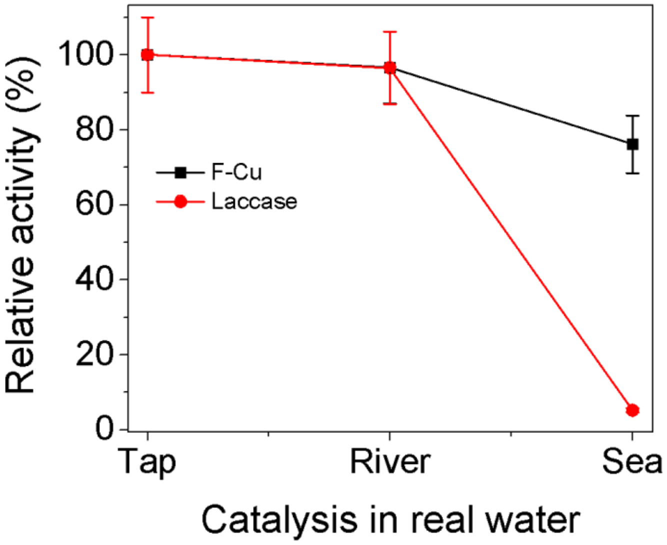

Supplementary Fig. 4. Relative catalytic activity of F-Cu bionanozyme in real water samples (tap water, river water, and seawater). N = 3 independent experiments, error bars represent standard deviations of three independent measurements.

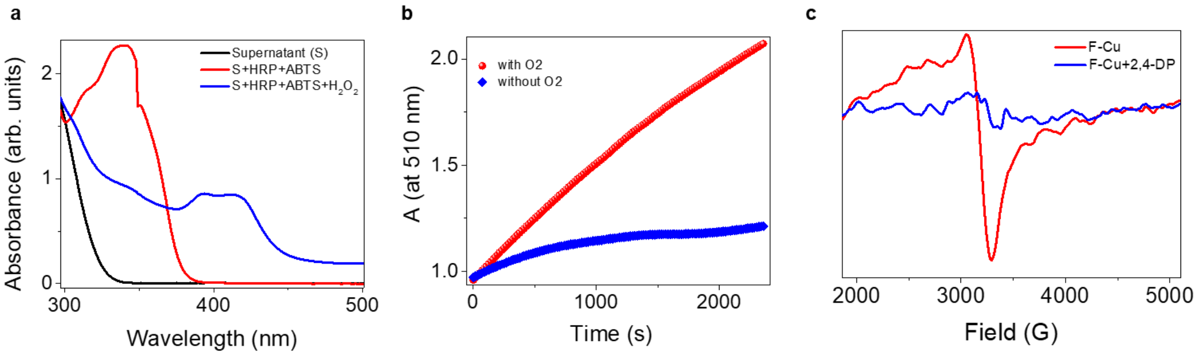

Supplementary Fig. 5. Evaluation of laccase-mimicking catalytic activity of F-Cu bionanozymes. **a**, UV-visible absorbance spectra of the control experiment testing the *in-situ* production of H_2O_2. **b**, UV-vis kinetic traces at 510 nm in the presence and absence of oxygen (O_2), Solutions were made anaerobic (without O_2) by bubbling water-vapor saturated nitrogen for 45 min. **c**, EPR spectra of F-Cu bionanozyme before and after addition of substrate 2,4-DP.

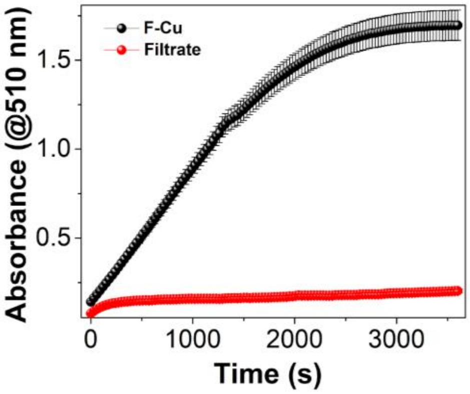

Supplementary Fig. 6. Progress of the catalytic 2,4-DP oxidation reaction in the presence of collected F-Cu and filtrate solution. (reaction conditions: F-Cu (1 mg/mL) was incubated in a standard reaction solution (PBS buffer (1X), pH 7.25) at 25 °C for 1800 s and then collected by centrifugation and ultrafiltration. Next, the activity of the filtrate (or leaching) solution was compared with that of the collected F-Cu under the same reaction conditions). The resulting reaction progress showed no significant catalytic activity in the filtrate solution; however, the observed activity is retained in the collected F-Cu bionanozyme. These results demonstrate that the observed catalytic activity mainly originates from the F-Cu bionanozyme themselves and not from any possible dissolved/released Cu^{2+} ions or non-assembled structures.

Supplementary Table 2. Comparison of relative specific catalytic activity of laccase mimicking nanozymes

**Nanozyme**	**Substrate**	**Conditions**	**Specific activity of nanozyme, U/mg**	**Specific activity of laccase, U/mg**	**Ratio of Specific activity of nanozyme to laccase**	**Ref.**
F-Cu	2,4-dichlorophenol & 4-aminoantipyrine	pH 7.2 C_{cat} = 0.1 mg/mL C_{sub} = 0.1 mg/mL	10.8	0.54	20	This work
CH-Cu[a]	2,4-dichlorophenol	pH 6.8 C_{cat} = 0.1 mg/mL C_{sub} = 0.1 mg/mL	0.012	0.031	0.39	Wang et al.[1]
Cu-CD[b]	*p*-phenylenediamine	pH 7 C_{sub} = 10 mM	0.2	0.5	0.4	Ren *et al.*[2]
Cu/GMP[c]	2,4-dichlorophenol & 4-aminoantipyrine	pH 6.8 C_{cat} = 0.1 mg/mL C_{sub} = 0.1 mg/mL	No data	No data	No data	Liang *et al.*[3]

[a]Dipeptide (Cys-His)-coordinated Cu complex

[b]Copper-containing carbon dots

[c]Guanosine monophosphate (GMP)-coordinated copper complex

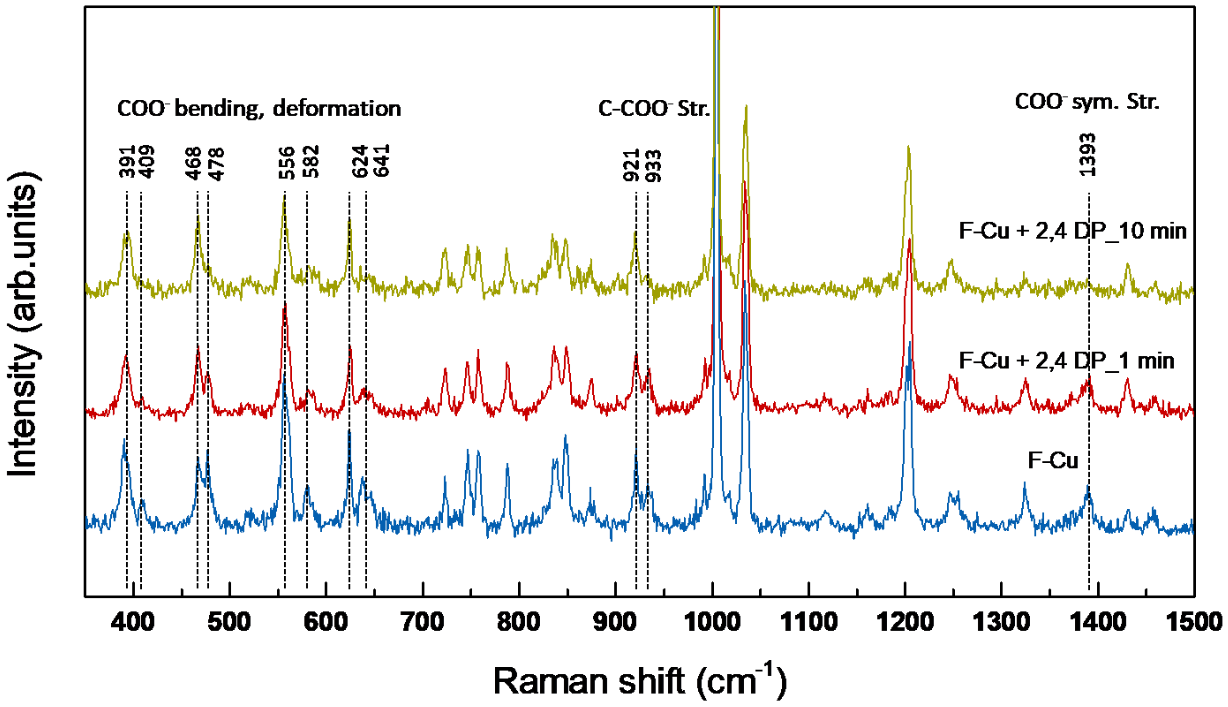

Supplementary Fig. 7. Time-dependent changes in the Raman spectrum of F-Cu after the addition of 2,4-DP. Various vibrational modes originating from the carboxylate ion (C-COO^-) such as COO^- bending, deformation, symmetric stretching (sym. str.), and C-COO^- stretching (str.) modes have been assigned. Note that the signal from 2,4-DP is absent in the spectra due to its low concentration (1 mM).

The Raman spectrum of the F-Cu catalyst before and after the addition of 2,4-DP is shown in Supplementary Fig. 7. The data below 1500 cm^{-1} is only shown here as no marked changes are observed in the higher wavenumber region. The vibrational modes originating from the carboxylate ion (C-COO^-) are identified based on the mode assignment given in earlier reports.[4–6] The most intense modes observed between 1000-1050 cm^{-1} and around 1200 cm^{-1} originate from the phenyl ring. The modes corresponding to the carboxylate group are important for the present discussion as they are undergoing significant changes upon 2,4-DP addition, while the phenyl ring modes do not exhibit any marked changes. Moreover, Cu^{2+} binding and catalytic oxidation are expected to occur at the C-COO^- site. The appearance of the doublet modes corresponding to COO^- bending, stretching, and deformation vibrations in the Raman spectrum of F-Cu catalyst is due to the coordination of phenylalanine (F) molecules with Cu^{2+} ions. Only one mode is observed that corresponds to C-COO^- stretching (at 1393 cm^{-1}) that may be due to the insufficient intensity of the second mode. When 2,4-DP is added to F-Cu, all the higher wavenumber modes of the doublet COO^- vibrations in F-Cu start to weaken in intensity as a function of reaction time. Some of these modes disappeared in the spectrum collected after 10 minutes of reaction. This indicates the weakening of Cu^{2+}

binding with the carboxylate ion, possibly indicating protonation of carboxylate groups in the F-Cu nanozyme.

Supplementary References

1. Wang, J. *et al.* Construction of a Bioinspired Laccase-Mimicking Nanozyme for the Degradation and Detection of Phenolic Pollutants. *Appl. Catal. B Environ.* **254**, 452–462 (2019).

2. Ren, X., Liu, J., Ren, J., Tang, F. & Meng, X. One-pot Synthesis of Active Copper-Containing Carbon Dots with Laccase-Like Activities. *Nanoscale* **7**, 19641–19646 (2015).

3. Liang, H. *et al.* Multicopper Laccase Mimicking Nanozymes with Nucleotides as Ligands. *ACS Appl. Mater. Interfaces* **9**, 1352–1360 (2017).

4. Madzharova, F., Heiner, Z. & Kneipp, J. Surface Enhanced Hyper-Raman Scattering of the Amino Acids Tryptophan, Histidine, Phenylalanine, and Tyrosine. *J. Phys. Chem. C* **121**, 1235–1242 (2017).

5. Nabiev, I. R., Savchenko, V. A. & Efremov, E. S. Surface-enhanced Raman Spectra of Aromatic Amino Acids and Proteins Adsorbed by Silver Hydrosols. *J. Raman Spectrosc.* **14**, 375–379 (1983).

6. Hernández, B., Pflüger, F., Kruglik, S. G. & Ghomi, M. Characteristic Raman Lines of Phenylalanine Analyzed by a Multiconformational Approach. *J. Raman Spectrosc.* **44**, 827–833 (2013).
