# Supplementary material for: Single amino acid bionanozyme for environmental remediation
Source: Nat Commun. 2022 Mar 21;13:1505. doi: 10.1038/s41467-022-28942-0 (PMC8938493; doi:10.1038/s41467-022-28942-0)
Supplement: Supplementary file 2 — Description of Additional Supplementary Files [file 41467_2022_28942_MOESM2_ESM.pdf]

Description of Additional Supplementary Files

File name: Supplementary Movie 1
Description: In-situ monitoring of the F-Cu crystallization process under an optical microscope.

File name: Supplementary Movie 2
Description: In-situ optical microscopy observation of phenylalanine-coordinated Copper ions (F–Cu) crystallization kinetics.

File name: Supplementary Movie 3
Description: In-situ reaction monitoring of 2,4-DP and 4-AP catalyzed by F-Cu crystals.

File name: Supplementary Movie 4
Description: Real-time monitoring of 2,4-DP and 4-AP oxidation in the presence and absence of the F-Cu catalyst.
